# Oral Glucose Tolerance Testing identifies HIV+ infected women with Diabetes Mellitus (DM) not captured by standard DM definition

**DOI:** 10.4172/2155-6113.1000545

**Published:** 2016-02-20

**Authors:** Sophie Seang, Jordan E Lake, Fang Tian, Kathryn Anastos, Mardge H Cohen, Phyllis C Tien

**Affiliations:** 1 Department of Medicine, Division of Infectious Diseases, University of California, Los Angeles, California, USA; 2 Anthem, Inc., Alexandra, VA, USA; 3 Department of Medicine and Epidemiology and Population Health, Albert Einstein College of Medicine and Montefiore Medical Center, Bronx, New York, USA; 4 Departments of Medicine, Cook County Health & Hospital System, and Rush University, Chicago, Illinois, USA

**Keywords:** HIV, Women, Diabetes mellitus, Oral glucose tolerance test

## Abstract

**Objective:**

HIV-infected (HIV+) individuals may have differential risk of diabetes mellitus (DM) compared to the general population, and the optimal diagnostic algorithm for DM in HIV+ persons remains unclear. We aimed to assess the utility of oral glucose tolerance testing (OGTT) for DM diagnosis in a cohort of women with or at risk for HIV infection.

**Methods:**

Using American Diabetic Association DM definitions, DM prevalence and incidence were assessed among women enrolled in the Women’s Interagency HIV Study. DM was defined by 2-hour OGTT ≥ 200 mg/dL (DM_OGTT) or a clinical definition (DM_C) that included any of the following: (i) anti-diabetic medication use or self-reported DM confirmed by either fasting glucose (FG) ≥126 mg/dL or HbA1c ≥ 6.5%, (ii) FG ≥ 126 mg/dL confirmed by a second FG ≥ 126 mg/dL or HbA1c 6.5%, or (iii) HbA1c 6.5% confirmed by FG ≥ 126 mg/dL cohort.

**Results:**

Overall, 390 women (285 HIV+, median age 43 years; 105 HIV−, median age 37 years) were enrolled between 2003-2006. Over half of all women were African American. Using DM_C, DM prevalence rates were 5.6% and 2.8% among HIV+ and HIV− women, respectively. Among HIV+ women, adding DM_OGTT to DM_C increased DM prevalence from 5.6% to 7.4%, a 31% increase in the number of diabetes cases diagnosed (p=0.02). In HIV− women, no additional cases were diagnosed by DM-OGTT.

**Conclusion:**

In HIV+ women, OGTT identified DM cases that were not identified by a standardized clinical definition. Further investigation is needed to determine whether OGTT should be considered as an adjunctive tool for DM diagnosis in the setting of HIV infection.

## Introduction

Since effective antiretroviral therapy (ART) has prolonged life expectancy among HIV-infected (HIV+) persons, non-AIDS events have become increasingly important causes of morbidity and mortality [1]. HIV+ persons on ART may have up to four-fold increased risk for diabetes mellitus (DM) compared to HIV-uninfected (HIV−) persons, with 2.5-7.0% reported DM prevalence rates [2,3]. Traditional DM risk factors such as older age, obesity, family history, black, Asian or Hispanic ethnicity [1,4] and HIV-related parameters (including ART exposure) have been associated with DM incidence in HIV+ persons [5,6].

Patients with undiagnosed/under-treated DM are at increasing risk for cardiovascular disease (CVD) due to the micro- and macro-vascular injuries that progressively develop. HIV+ persons are also at increased CVD risk [7]. This, combined with the elevated risk of glucose disorders and rapid aging of the HIV+ patient population, amplifies the need for early and accurate DM diagnosis to minimize CVD risk in this population.

The American Diabetes Association (ADA) guidelines recommend fasting plasma glucose (FPG), glycosylated hemoglobin (HbA1c) and two-hour, 75g oral glucose tolerance testing (OGTT) for DM diagnosis [8]. In HIV+ persons, HbA1C may underestimate glycemia [9-11], possibly as a result of low-grade hemolysis [10,11]. OGTT has not commonly been used for DM diagnosis in large HIV+ cohorts [2,3], likely due to a combination of cost, performance time, low reproducibility and high intra-individual variability [12]. Nevertheless, in the general population, OGTT has been reported to improve DM diagnosis in older individuals [13] and to be more predictive of CVD than FPG [14]. Overall, controversies regarding the optimal diagnostic algorithm for DM in HIV+ persons persist.

We examined the prevalence and incidence of DM in participants of the Women’s Interagency HIV Study (WIHS) over five years of follow-up and aimed to determine the utility of OGTT for DM diagnosis in women with and without HIV. The WIHS has previously demonstrated that while HIV-infected women have an increased DM risk [5,6,15], a DM diagnosis made without any confirmatory criteria overestimated DM incidence. By contrast, the use of confirmatory criteria (including an elevated A1C) increased the diagnostic accuracy and slightly attenuated the magnitude of the association otherwise observed between HIV and DM [15]. We now build upon our prior work by investigating how the use of OGTT might impact on DM prevalence and incidence in HIV+ and HIV− women compared to when DM is diagnosed using confirmatory criteria including AIC.

## Patients and Methods

### Study population

The WIHS is a multicenter, prospective cohort study established in 1994 to investigate the natural history of women living with or at risk for HIV. The methods and baseline characteristics of WIHS participants have been described previously [16]. Between April 2003 and March 2006, a subset of 440 WIHS women from the San Francisco, Bronx and Chicago sites were enrolled into a prospective metabolic study (MS). Exclusion criteria included: age >65 years old; weight >264 pounds and height >6 feet and 1 inch (as per the dual X-ray absorptiometry manufacturer criteria); pregnancy or breastfeeding in the past six months; use of female hormones, growth hormone or steroids in the last year; and HIV seroconversion in the year prior to MS entry or during follow-up. All eligible participants underwent baseline, two-year and five-year follow-up visits for metabolic disease outcomes. For two- and five-year incidence analyses, women with prevalent DM at baseline were excluded. Informed consent was obtained from all participants in accordance with the US Department of Health and Human Services guidelines and the institutional review boards of each participating institution.

### Outcome definitions

DM was defined according to ADA criteria [8] and two DM groups were created: 1) DM defined by two-hour OGTT ≥ 200 mg/dL (DM_OGTT) or 2) DM defined using a clinical definition (DM_C). The latter included any of the following criteria: (i) anti-diabetic medication use or self-reported DM confirmed by either FPG ≥ 126 mg/dL or HbA1c ≥ 6.5%, (ii) FPG ≥ 126 mg/dL confirmed by a second FPG ≥ 126 mg/dL or HbA1c ≥ 6.5% or (iii) HbA1c ≥ 6.5% confirmed by FPG ≥ 126 mg/dL. Women were considered to have prevalent DM by clinical definition if they met any of the above criteria at and prior to baseline, and incident DM _C if they met any of the above criteria at two- or five-year follow-up. The primary and secondary endpoints were the number of prevalent DM cases and incident DM cases over five years, respectively, diagnosed by each DM criteria.

### Statistical methods

Differences between HIV+ and HIV− women were compared using the chi-squared test for binary or categorical variables, the t-test for continuous and normally distributed variables or the Wilcoxon rank sum test for continuous variables that were not normally distributed. DM prevalence by HIV serostatus was analyzed using the chi-squared test. McNemar’s test was performed to compare DM prevalence between the two outcome definitions (DM_OGTT vs. DM_C). DM incidence by HIV serostatus over five years of follow-up was analyzed using the chi-squared test. Multivariate pooled logistic regression was performed to study associations between HIV serostatus, DM and the covariates [including age, race/ethnicity, family history of DM, body composition, hepatitis C virus (HCV) co-infection, current smoking and menopausal status; hematocrit and mean corpuscular volume were added in DM_C model]. Covariates included in the final multivariate model were based upon either significance in the crude model or clinical consideration and/or face validity. Estimated adjusted hazard odds ratios (OR) are reported, and a p value ≤0.05 defined statistical significance.

## Results

### Population

Among 418 women with available OGTT data enrolled in the MS, 390 women (285 HIV+, 105 HIV−) met criteria for inclusion in the analysis of DM prevalence. Baseline demographic and clinical characteristics are described in Table 1. Over half of women were African American. HIV+ women were older (median 43 years vs. 37 years, p <0.0001) and more likely to be post-menopausal (32% vs. 7%, p<0.0001) and have HCV co-infection (33% vs. 15%, p=0.0005) than HIV− women. Compared to HIV− women, HIV+ women also had lower body mass index (BMI), smaller hip and waist circumferences and less trunk and leg fat, but greater triglyceride levels and lower high-density lipoprotein cholesterol levels. Among HIV+ women, median CD4+ T lymphocyte and HIV-1 RNA values were 400 cells/mm^3^ and 590 copies/mL, respectively. The cumulative years of ART exposure to the PI, NRTI and non-NRTI classes were 2.5, 5.5 and 1.5 years, respectively.

### Baseline DM prevalence

**Figure 1:**
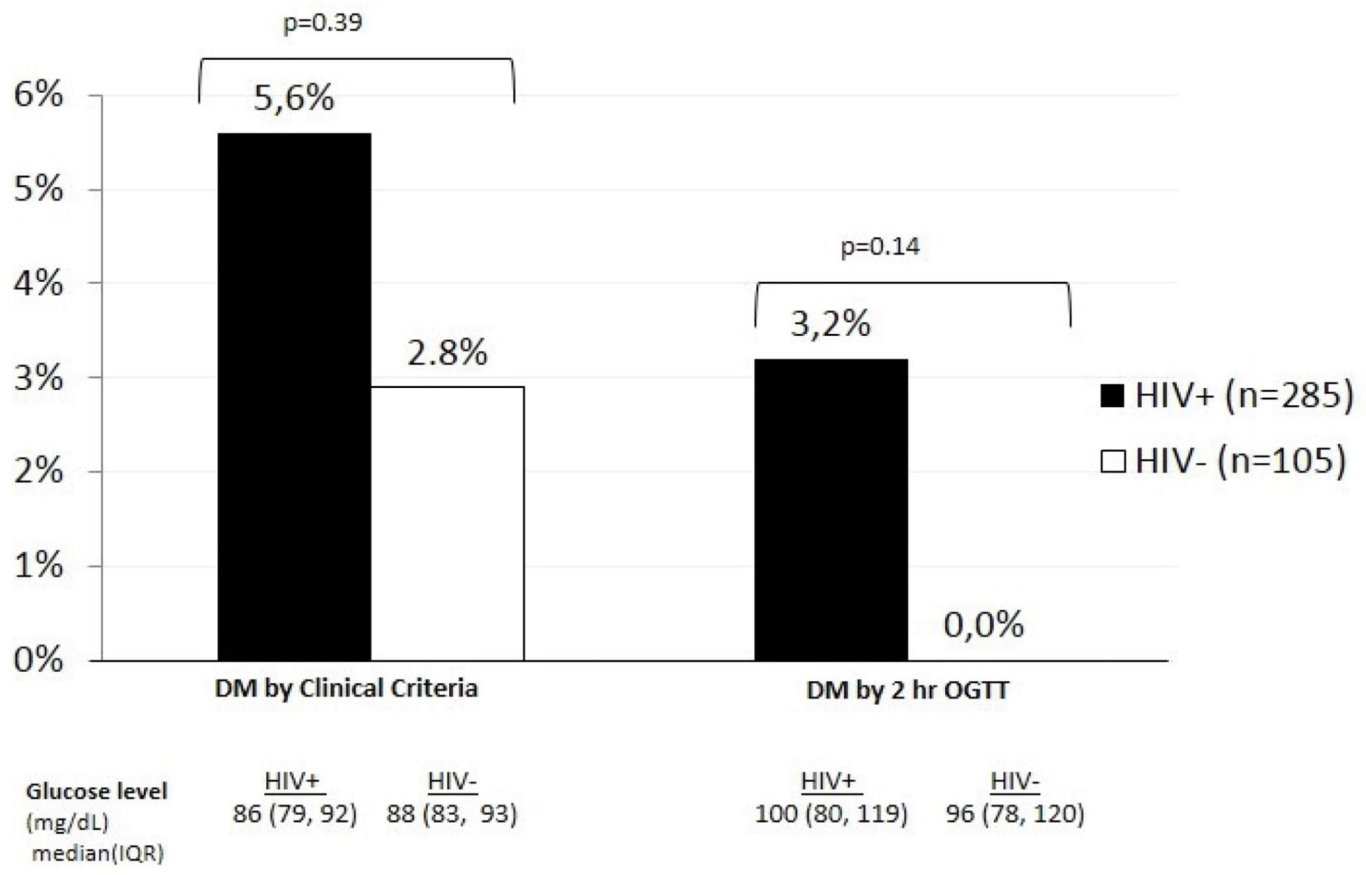
Prevalence of diabetes mellitus using clinical criteria and using 2-hour OGTT by HIV status. DM: diabetes mellitus; OGTT: oral glucose tolerance testing ■ HIV+ (n=285) □ HIV− (n=105)

Using DM_C, DM prevalences were 5.6% and 2.8% among HIV+ and HIV− participants, respectively. Using DM_OGTT, 3.2% of women met DM diagnostic criteria, all of whom were HIV+ (Figure 1). HIV+ women had slightly lower median FGP levels than HIV− women (86 mg/dL vs. 88 mg/dL; p=0.08). After OGTT, median glucose values were slightly higher in HIV+ women but the difference was not significant (100 mg/dL vs. 96 mg/dL; p=0.25).

Among HIV+ diabetic women (diagnosed by DM_C or DM_OGTT definition, n=21), 24% (5/21) were diagnosed only by the OGTT definition and not by the standard clinical definition. Combining OGTT and clinical criteria for DM diagnosis increased DM prevalence from 5.6% (16/285) to 7.4% (21/285) among HIV+ women, which represents a 31% (5/16) relative increase in the number of DM cases diagnosed vs. DM_C alone (p=0.02). No significant differences in clinical or demographic characteristics were reported between HIV + women diagnosed with DM by DM_C with normal OGTT (n=9) vs. DM_OGTT without meeting DM_C (n=5). Additionally, there were no statistically significant clinical or demographic differences between non-diabetic, HIV+ women with normal OGTT (n=230) and HIV+ DM_OGTT cases (n=5). Among HIV− women, three DM cases were diagnosed by the DM_C definition, and all of them had normal OGTT values.

### DM incidence over five-year follow up and factors associated with DM incidence

A total of 303 women (215 HIV+, 88 HIV−) were included in the five-year analysis for incident DM. The clinical characteristics were similar to the overall group described in Table 1. The five-year DM incidence rate among all women, using DM_C definition and DM_OGTT criteria was 7.0% and 4.0%, respectively (p=0.21). Among all women, greater leg fat was associated with lower odds of incident DM (DM_C: OR 0.76, 95% CI 0.58-0.99, p=0.04; DM_OGTT: OR 0.75, 95% CI 0.60-0.95, p=0.02), and greater trunk fat with greater odds of incident DM (DM_C: OR 1.22, 95% CI 1.04-1.43, p=0.01; DM_OGTT: OR 1.20, 95% CI 1.05-1.38, p=0.01). In a subset analysis of DM cases among HIV+ women, these associations remained significant.

## Discussion

In our study, nearly one quarter of HIV+, diabetic women were diagnosed with DM by the OGTT criteria alone and not by a standardized clinical DM definition. Adding the OGTT criteria for DM diagnosis enabled a 31% relative increase in the number of prevalent DM cases diagnosed among HIV+ women. Although the overall prevalence of DM in our HIV+ population remains low (5.6% to 7.4%), these results highlight the potential utility of OGTT among HIV+ individuals.

Previous studies have reported increased prevalence rates of DM in HIV+ individuals, but few have reported DM prevalence within the same cohort using different algorithms. Supporting our findings, Howard et al. [17] reported (in a cohort of middle age women living with or at risk for HIV infection) that 23% of women with DM defined by OGTT had FPG <126 mg/dL and Gianoti et al. [18] reported that 4% of HIV+ men with normal FPG levels, had DM diagnosed by OGTT alone. While our conclusion was different from an earlier study in the WIHS, [19] that study did not specifically address whether the use of OGTT would detect additional cases of DM in HIV-infected adults; rather that study examined the association of HIV with prevalent DM using a DM definition that included elevated fasting plasma glucose and/or an OGTT finding consistent with DM. Because that study found little difference in DM prevalence between HIV-infected and uninfected women, they concluded that there was limited value in using OGTT in the HIV setting.

OGTT was first introduced for DM diagnosis in 1980 by the World Health Organization [20] with supporting epidemiological evidence that OGTT increased the number of DM diagnoses in the general population, particularly in patients >50 years old [13]. Physiologically, diabetes development involves impaired insulin sensitivity and insulin secretion that first affects post-prandial glycemia, while chronic fasting glycemia may temporarily remain normal [21]. Over time, the extent and magnitude of post-prandial hyperglycemia leads to the chronic, sustained hyperglycemia associated with DM complications [22]. This sequential progression of glucose abnormalities may explain discordant results between OGTT and FPG in clinical studies. Additionally, the absence of discordance among HIV− women may be explained by the low prevalence of DM in this group. Our findings also showed that 9 of 21 prevalent DM cases would have not been identified by OGTT alone. These results suggest that further investigation is needed to determine whether OGTT should be considered as an adjunctive tool for DM diagnosis in the setting of HIV infection including whether OGTT should be performed in HIV+ patients with specific risk factors, such as obesity, especially as OGTT is expensive and time-consuming.

To determine if the use of OGTT could be more relevant in a specific HIV+ subgroup, we compared clinical characteristics of diabetic, HIV+ women with non-diabetic, HIV+ women. Our analysis did not show significant differences in demographic or clinical characteristics between HIV+, non-diabetic women compared to HIV+, diabetic women, a finding that may result from the low number of participants with prevalent DM. However, HIV+, non-diabetic women tended to have more leg fat than HIV+, diabetic women diagnosed either by OGTT or DM_C criteria (p=0.10 for both). In our multivariate model, we found that greater leg fat was significantly associated with decreased odds of DM, whereas greater trunk fat increased the odds of DM defined by OGTT definition among HIV+ women. These results are consistent with previous data in HIV+ persons in which lipodystrophy was associated with incident DM. Kosmiski et al. [23] showed that greater trunk subcutaneous adipose tissue and less leg subcutaneous adipose tissue were significantly associated with higher 2-hour glucose values in HIV+ persons. Adipose tissue is an endocrine organ with a determinant role in the secretion of the adipokines that control insulin sensitivity. In HIV+ individuals with significant lipoatrophy, perturbations of adipokine homeostasis may contribute to the development of insulin resistance [24]. Although our dual x-ray absorptiometry data cannot accurately distinguish subcutaneous from visceral adipose tissue, our results support the hypothesis that regional adiposity is related to insulin sensitivity in HIV infection. Considering the interaction between lipodystrophy and insulin sensitivity in HIV+ persons, OGTT could be a useful DM diagnostic tool in subjects with abnormal fat distribution. Additionally, in a study among HIV+ overweight women, Danoff et al. [19] reported BMI as a significant parameter for prediction of DM. Overall, DM screening among HIV+ women using OGTT could be more relevant in individuals at risk for CVD (PI exposed or with traditional CV risk factors) or with metabolic disorders.

This study has several strengths, including our ability to assess DM risk longitudinally, access to a well-characterized cohort of HIV+ and HIV− women, and the use of standardized definitions for DM. The latter is particularly important, as most previous studies in HIV have defined DM by self-report or FPG levels alone. Our study also has several limitations. First, 17% (n=63/366) of participants were lost-to-follow up over the five-year period. Moreover, the small number of incident DM cases in HIV+ women may have limited our ability to detect associations between risk factors and DM outcomes. Lastly, our data cannot be generalized to HIV+ men.

In conclusion, the prevalence and five-year incidence of diabetes among HIV+ and HIV− women were consistent with previously reported rates. DM diagnosis by OGTT criteria identified DM cases among HIV+ women that would not have been diagnosed by the standardized clinical definition alone. Further research is needed to identify the subgroup of HIV+ women most likely to benefit from OGTT.

## Figures and Tables

**Table 1:** Baseline clinical and demographic characteristics of 390 women by HIV status.

	HIV+	HIV−	p-value
	n=285	n=105	
Age (years) ^+^ *median (IQR)*	43 (38, 49)	37 (32, 44)	<.0001
Race			0.56
American African	58%	57%	
Hispanic	27%	31%	
Caucasian/Other	15%	11%	
Cohort*			<.0001
1994/1995	65%	41%	
2001/2002	35%	59%	
Current Smoker	59%	69%	0.10
Post-menopausal*	32%	7%	<.0001
DM family history	29%	39%	0.06
Chronic HCV infection*	33%	15%	0.0005
Body measures, *median (IQR)*			
BMI (kg/m^2^)^+^	27 (23, 31)	30 (26, 36)	<.0001
Hip circumference (cm) ^+^	98 (91, 108)	105 (97, 116)	<.0001
Waist circumference (cm) ^+^	88 (80, 98)	93 (83, 103)	0.02
Trunk fat (kg) ^+^	13 (9, 17)	16 (11, 22)	0.0003
Leg fat (kg) ^+^	9 (6, 13)	12 (8, 16)	<.0001
Lipids, *median (IQR)*			
HDL (mg/dL)^+^	43 (35, 54)	50 (43, 63)	<.0001
LDL (mg/dL)	94 (74, 113)	101 (75, 124)	0.07
TG (mg/dL)^+^	109 (77, 158)	92 (63, 129)	0.002
CD4+ count (/mm3), *median (IQR)*	400 (257, 585)		
CD4 nadir (/mm3), *median (IQR)*	247 (128, 350)		
HIV-1 RNA (copies/mL), *median (IQR)*	590 (80, 8900)		
AIDS diagnosis	46%		
On ART since last visit	61%		
PI	55%		
NRTI	99%		
NNRTI	44%		
Years on ART, *median (IQR)*	5 (1, 10)		
PI	2.5 (0, 4.5)		
NRTI	5.5 (3, 8)		
NNRTI	1.5 (0, 3)		

Percentage or median with interquartile range shown

* *P*<*0.05 for Χ^2^ test comparing HIV*− *versus HIV*+ *group*

+ *P*<*0.05 for Wilcoxon rank sum test comparing HIV*− *versus HIV*+ *group*

DM: diabetes mellitus; HCV-RNA: hepatitis C virus; BMI: body mass index; HDL: high density lipoprotein; LDL: low density lipoprotein; TG: triglyceride; HAART: highly active antiretroviral therapy; PIs: protease inhibitors; NNRTIs: Non-nucleoside reverse transcriptase inhibitors; NRTI: nucleoside reverse transcriptase inhibitors.
